# OPN gene polymorphisms influence the risk of knee OA and OPN levels in synovial fluid in a Chinese population

**DOI:** 10.1186/ar4129

**Published:** 2013-01-05

**Authors:** Yongqing Jiang, Meng Yao, Qingpeng Liu, Changwei Zhou

**Affiliations:** 1 Department of Orthopedics, The Second Affiliated Hospital of Harbin Medical University, 194 Nangang District, Harbin, 150081, China

## Abstract

**Introduction:**

A body of studies suggests the role of osteopontin (OPN) in onset and development of osteoarthritis (OA), however, the association between OPN polymorphisms and OA susceptibility as well as its clinical features has not been reported.

**Methods:**

A total of 750 patients with primary knee OA and 794 healthy volunteer were enrolled as controls. Both OA and control groups were interviewed to obtain demographic and clinical data. Three polymorphisms of OPN gene, namely, -156GG/G, -443C/T and -66T/G were determined. The levels of the full length and the thrombin-cleaved OPN in synovial fluid (SF) from OA subjects were measured.

**Results:**

We found the polymorphisms of the -443C/T and the -66/T/G were significantly associated with the OA risk and the radiographic severity. The -443TT and -66GG showed protective effect against developing OA and were associated with lower Kellgren-Lawrence grade. Besides, the polymorphisms of -443C/T and -66T/G significantly affected the thrombin-cleaved OPN levels in SF from OA subjects. Subjects with -443TT and -66GG genotypes had lower thrombin-cleaved OPN levels in SF. The thrombin-cleaved OPN levels in SF were positively correlated to the radiographic severity of OA.

**Conclusions:**

Our findings suggest that certain OPN gene polymorphisms may be used as molecular markers for the susceptibility and severity of OA.

## Introduction

Osteoarthritis (OA) is a degenerative joint disorder resulting in substantial morbidity and disability in the elderly [1,2]. It is now accepted that the excessive, spontaneous production of cytokines and mediators of inflammation plays a significant role in the molecular pathogenesis of OA, contributing to a highly catabolic state, chondrocyte apoptosis, and the resultant progressive degeneration of articular cartilage [3-5]. The etiology of OA is largely unknown. Aging, trauma, hormonal and mechanical factors are reported to contribute to the onset and progression of OA [6-8]. Inheritance studies involving family groups and twin pairs have demonstrated that a large proportion of OA cases can be attributed to genetic factors, suggesting that the etiology of OA clearly has a genetic component [9,10]. In recent years, a number of gene polymorphisms involved in development of knee OA have been identified, such as those localized in, or adjacent to the encoding sequences for the vitamin D receptor [11], estrogen receptor alpha [12] or calcitonin [13].

Osteopontin (OPN) is a matricellular protein that through interactions with its receptors, integrins α4β1, α9β1, αv (β1, β3, β5), and CD44 variants, participates in a wide range of physiologic and pathologic processes, including wound healing, bone turnover, tumor genesis, inflammation, and immune responses [14-17]. The association between OPN and joints had been studied. OPN expression during chondrocyte maturation is one of the important events involved in cartilage-to-bone transitions, and OPN is reportedly involved in the molecular pathogenesis of OA, contributing to progressive degeneration of articular cartilage [18]. Clinically, OPN in plasma and synovial fluid (SF) has been reported to be related to progressive joint damage in knee OA, suggesting that OPN may serve as a biochemical marker for determining disease severity [18].

The expression of OPN is significantly influenced by genetic polymorphisms of its promoter [19]. Several polymorphisms in the human OPN encoding gene have been identified in different populations, of which the -156 GG/G, -443C/T and -66T/G polymorphisms were mostly studied. These OPN genetic polymorphisms have been reported to be associated with inflammatory disease, such as systemic lupus erythematosus [20], chronic hepatitis C [21], lupus nephritis [22], and large artery atherosclerosis [23].

Although a body of studies suggests the role of OPN in the onset and development of OA, the association between OPN polymorphisms and OA susceptibility, as well as its clinical features, has never been reported. We carried out a case-control study to clarify whether the abovementioned genetic polymorphisms in the OPN gene are associated with the susceptibility and severity of OA in a Chinese cohort.

## Materials and methods

### Patients

A total of 750 patients with primary OA of the knee were recruited from February 2007 to December 2011. The diagnosis of knee OA was based on the American College of Rheumatology criteria [24]. The severity of OA was evaluated by Kellgren-Lawrence (KL) grade. We enrolled 794 healthy volunteers as controls. Both the OA and control groups were interviewed to obtain demographic data and information on all of the established risk factors. Patients with other etiologies causing knee diseases, such as inflammatory arthritis (rheumatoid, polyarthritic, or autoimmune disease), post-traumatic or post-septic arthritis, skeletal dysplasia or developmental dysplasia were excluded from the OA group.

All the controls were enrolled in this study based on the following: 1) no history, or signs or symptoms of arthritis or joint diseases (pain, swelling, tenderness, or restriction of movement) and 2) normal appearances on radiography of the knee. The clinical characteristics of all enrolled subjects, including age, sex, body mass index (BMI), smoking status, knee activity and regular exercise were recorded. Obesity was defined as BMI > 30 kg/M^2^. The study was approved by the ethics review committee of Harbin Medical University and written informed consent was obtained from all participants.

### *OPN *gene polymorphisms

DNA was extracted from peripheral whole blood using a Qiagen DNA Isolation Kit (Qiagen, Valencia, CA, USA). Three single nucleotide polymorphisms on the promoter region of *OPN *gene, including -66T/G (rs28357094), -156G/GG (rs17524488), and -443C/T (rs11730582), were determined using TaqMan 5' allelic discrimination assay. It was performed using a commercially available kit, Assays-on-DemandTM single nucleotide polymorphism (SNP) genotyping products (Applied Biosystems, Foster City, CA, USA). SNP amplification assays were used according to the manufacturer's instructions. In short, 10 ng of sample DNA in 25 μL of reaction solution, containing 12.5 μL of the 2× TaqMan^® ^Universal PCR Mix (Applied Biosystems), and 1.25 μL of pre-developed assay reagent from the SNP genotyping product containing two primers and two MCB-Taqman probes. The reaction condition consisted of pre-incubation at 50°C for 2 minutes, and at 95°C for 10 minutes, followed by 40 cycles of 95°C for 15 s, and 60°C for 1 minute. Amplifications were performed in an ABI Prism^® ^7500 Sequence Detection System (Applied Biosystems). To reconfirm the results of the genotypes, we randomly selected 25 patients and 25 controls (10% of the study subjects) for re-genotyping by direct sequencing; the results were 100% identical.

### OPN level detections

SF was obtained from 175 OA patients; none was obtained from controls due to ethical concerns. The levels of the full length and the thrombin-cleaved OPN in SF were determined by capture ELISA according to the protocol provided by the manufacturer (Calbiochem, San Diego, CA, USA). The sensitivity for OPN was 3.33 ng/ml, with intra- and inter-assay coefficients of variation (CV) of < 5%, and < 10%, respectively [25].

### Western blot analysis

A 5-μL aliquot from each sample of SF was subjected to SDS-PAGE; the separated proteins were electro-transferred onto polyvinylidene fluoride membranes (Millipore, Bedford, MA, USA). Nonspecific proteins on the membranes were blocked in 5% skimmed milk powder in PBS (thrombin-cleaved OPN overnight at 4°C. Immunoblotting was performed with use of the rabbit against human Full length OPN Anti-Osteopontin antibody (ab8448, 1:1,000 dilution, Abcam, Cambridge, MA, USA) and thrombin-cleaved OPN antibodies (Mouse Anti-Human Osteopontin N-Half Monoclonal, 1:1,000 dilution, Cosmo Bio Co., Ltd., Toyo, Japan). The membranes were then incubated with the appropriate horseradish peroxidase (HRP)-conjugated secondary antibodies (1:2,500 dilution). Immunoreactive proteins were visualized with the western blotting luminol reagent (Santa Cruz Biotechnology, Santa Cruz, CA, USA).

### Statistical analyses

Chi square (χ2) tests were used to compare genotype frequency and demographic distributions between cases and controls. Multiple logistic regression analysis was used to evaluate if each SNP was independently associated with OA when adjusted for the potential confounding effects of important clinical variables. The odds ratios (OR) and 95% CIs were calculated. The associations between the *OPN *haplotypes and OA risk were analyzed. The *D*' value and *r*2 for the three SNPs that we studied were calculated using SHEsis software [26]. All other analyses were performed using SPSS software (Statistical Package for the Social Sciences, version 16.0, SPSS Inc, Chicago, IL, USA).

## Results

Table 1 shows demographic and clinical characteristics of all subjects in the study. There were no significant differences in sex, age, smoking status, history of work involving heavy labor, hypertension, or diabetes mellitus between knee OA cases and controls. The OA patient group had a markedly higher BMI than the controls (*P *= 0.001).

**Table 1:** Demographic and clinical characteristics of all subjects in the study

Variables	Cases (*n *= 750)	Control (*n *= 794)	*P*
Age, years, mean ± SD	65.2 ± 7.6	65.1 ± 5.4	ns
Female, n (%)	401 (53.47%)	406 (51.13%)	ns
BMI, kg/m^2^, mean ± SD	27.7 ± 2.4	22.5 ± 3.1	0.001
Smoker, n (%)	283 (37.73%)	291 (36.65%)	ns
Family history of OA, n (%)	60 (8.00%)	67 (8.43%)	ns
History of labor work, n (%)	140 (18.66%)	158 (19.89%)	ns
Hypertension	198 (26.4%)	206 (25.9%)	ns
Diabetes mellitus	152 (20.3%)	166 (20.9%)	ns
KL grade			
= < 3	351	-	
> 3	399	-	

BMI, body mass index; OA, osteoarthritis; KL, Kellgren-Lawrence; ns, not significant.

Table 2 describes the genotype distributions and allele frequencies of *OPN *polymorphisms in OA and control subjects. The genotype frequencies for all polymorphisms did not differ significantly from those expected under Hardy-Weinberg equilibrium (both *P *> 0.05). The genotype and allele frequencies at *OPN *-156/G/GG were similar in OA and control subjects. In contrast, the -443C/T and -66/T/G genotype were significantly different between knee OA subjects and controls. The OA patients had significantly lower rates of -443TT and -66GG than controls (*P *< 0.001 and *P *= 0.013, respectively). Accordingly, the -443T and -66G allele frequencies were lower in OA patients than in controls (*P *= 0.018 and *P *= 0.009, respectively). Logistic regression analysis showed a significantly decreased risk for knee OA for the -443TT genotype compared with the -443CC genotype (OR 0.525, *P *< 0.001) after adjustment for sex, age, smoking status, family history and history of heavy labor. The adjusted OR for the -443T allele carriage was 0.689 (*P *< 0.001). Similarly the -66GG genotype carriers had a lower risk for OA, as the adjusted OR was 0.652 (*P *= 0.017) compared with the -66TT genotype. The adjusted OR for the -66G allele carriage was 0.815 (*P *= 0.011).

**Table 2:** Genotype distributions and allele frequencies of OPN polymorphisms in OA and control subjects

OPN polymorphism	Allele frequency	OA patients, n	%	Controls, n	%	Global *P*-value	Adjusted OR	95% CI		Adjusted *P*-value
-156/G/GG	GG	203	27.07	213	26.83	0.557	1			
	GGG	352	46.93	391	49.24		0.956	0.732	1.276	0.701
	GGGG	195	26.00	190	23.93		1.787	0.887	1.552	0.674
	G	758	50.53	817	51.45	0.318	1			
	GG	742	49.47	771	48.55		1.038	0.785	1.267	0.658
-443C/T	CC	167	22.27	119	14.99	< 0.001	1			
	CT	384	51.20	405	51.01		0.763	0.457	0.998	0.003
	TT	199	26.53	270	34.01		0.525	0.392	0.872	< 0.001
	C	718	47.87	643	40.49	0.018	1			
	T	782	52.13	945	59.51		0.689	0.575	0.896	< 0.001
-66T/G	TT	188	25.07	153	19.27	0.013	1			
	TG	369	49.20	402	50.63		0.738	0.516	0.988	0.035
	GG	193	25.73	239	30.10		0.657	0.494	0.874	0.017
	T	745	49.67	708	44.58	0.009	1			
	G	755	50.33	880	55.42		0.876	0.765	0.876	0.011

OPN, osteopontin; OA, osteoarthritis; OR, odds ratio.

The associations between the *OPN *haplotypes and knee OA risk were analyzed in this study. The D' value for the three SNPs were calculated with the SHEsis software [26]. All three SNPS were in strong linkage disequilibrium (LD) (all *D*' > 0.8). The estimated haplotype frequencies of the *OPN *SNPs are shown in Table 3. The haplotype G_-156_T_-443 _G_-66 _represented a protective effect for developing knee OA (adjusted OR 0.754, *P *= 0.005). Meanwhile, the GG_-156_C_-443 _T_-66 _showed a higher risk for developing OA (OR 1.943, *P *< 0.001).

**Table 3:** Associations between OPN haplotypes and risk of knee OA

Haplotype analysis*							
-156G/GG	-443C/T	-66T/G	Cases (frequency)	Controls (frequency)	Chi^2^	Pearson's *P*	Odds ratio (95% CI)
G	C	T	166	194.	4.451	0.065	0.789 (0.633, 1.092)
G	T	G	218	262	8.044	0.005	0.754 (0.620, 0.917)
G	C	T	209	206	0.206	0.650	0.953 (0.775, 1.172)
G	T	G	154	177	3.422	0.064	0.806 (0.642, 1.013)
GG	C	T	273	145	37.47	0.000	1.943 (1.566, 2.409)
GG	C	G	222	165	6.324	0.012	1.317 (1.062, 1.633)
GG	T	T	231	210	0.181	0.670	1.045 (0.854, 1.278)

*All haplotypes with frequency < 0.03 are ignored in this analysis.

We analyzed the genotype and the radiographic severity of OA in the patient group, who were grouped into subjects with KL grade = < 3 and those with KL grade > 3 (Table 3). We found that the -443C/T and -66T/G genotypes were significantly different between OA subjects with KL grade = < 3 and those with KL grade > 3 (both *P *< 0.001). Logistic regression analysis showed that the -66GG genotype and -443TT carriers were less likely to have severe OA (KL grade > 3) compared with -66TT and -443CC carriers. The adjusted OR for -443TT carriers was 0.529 (OR 0.664, *P *= 0.011, with -443CC as the reference) and the adjusted OR for -66GG carriers was 0.635 (OR 0.757, *P *= 0.023, with -66TT as the reference). The carriage of -443T and -66G represented a protective role against developing severe OA (adjusted OR 0.659 and 0.732, *P *= 0.008 and 0.017, respectively) (Table 4). The genetic polymorphisms of -156G/GG did not influence the severity of OA.

**Table 4:** Genotype and the radiographic severity in patients with OA

Genotype		KL = < 3, number of patients	%	KL > 3, number of patients	%	Global *P*-value	Adjusted OR	95% CI		Adjusted *P*-value
-156/G/GG	GG	92	26.21	91	22.81	0.462	1			
	GGG	166	47.29	206	51.63		0.787	0.564	1.178	0.223
	GGGG	93	26.50	102	25.56		0.916	0.638	1.376	0.653
	G	350	49.86	388	48.62		1			
	GG	352	50.14	410	51.38		0.934	0.786	1.186	0.648
-443C/T	CC	88	25.07	79	19.80	< 0.001	1			
	CT	176	50.14	188	47.12		0.816	0.537	1.264	0.352
	TT	87	24.79	132	33.08		0.664	0.365	0.879	0.011
	C	352	50.14	346	43.36		1			
	T	350	49.86	452	56.64		0.659	0.647	0.948	0.008
-66T/G	TT	97	27.64	91	22.81	0.462	1			
	TG	158	45.01	191	47.87		0.865	0.587	1.234	0.174
	GG	96	27.35	117	29.32		0.757	0.438	0.944	0.023
	T	332	47.29	373	46.74		1			
	G	370	52.71	425	53.26		0.7326	0.668	0.973	0.017

KL, Kellgren-Lawrence grade

**Figure 1:**
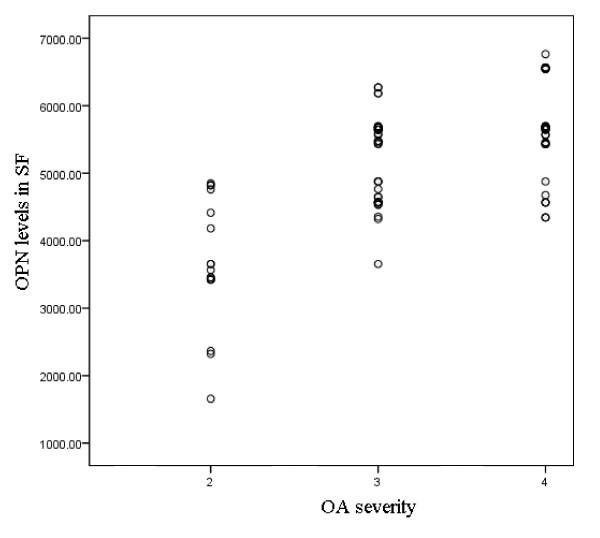
**Mean thrombin-cleaved osteopontin (OPN) levels in synovial fluid (SF) IN patients with osteoarthritis (OA) according to Kellgren-Lawrence (KL) grade**.

We further analyzed the correlation between the OPN level in SF and the radiographic severity of OA. The mean full length OPN levels were similar among KL grade 2, KL grade 3 and KL grade 4 subgroups (data not shown). In contrast, the mean thrombin-cleaved OPN levels in OA subjects were quite different among these KL-graded subgroups. The mean thrombin-cleaved OPN level was, 669.2 ± 476 (mean ± SD) pg/ml in patients with KL grade 2 OA, 5,203.6 ± 385 pg/ml in those with KL grade 3, and 5,642.7 ± 350 pg/ml in those with KL grade 4 (Figure 1). Positive correlation was found between the thrombin-cleaved OPN levels (but not the full length OPN levels) in SF and OA severity (Pearson correlation coefficient 0.692, *P *< 0.001).

**Figure 2:**
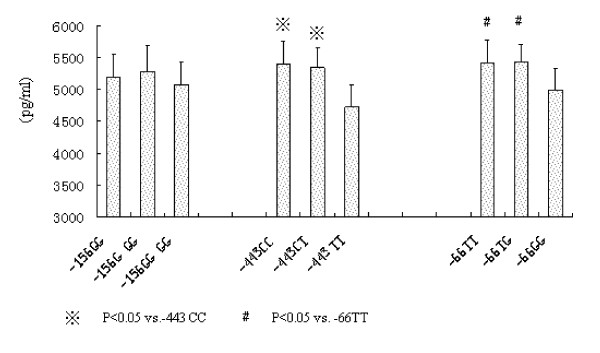
**Mean thrombin-cleaved osteopontin (OPN) levels in synovial fluid (SF) according to the genotypes of OPN gene polymorphisms**.

**Figure 3:**
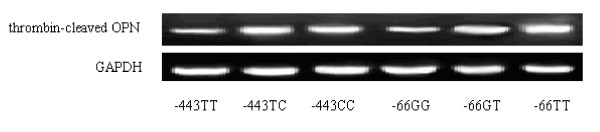
**Thrombin-cleaved osteopontin (OPN) levels in synovial fluid (SF) from different genotype carriers**. GAPDH, glyceraldehyde-3-phosphate dehydrogenase.

We next analyzed the thrombin-cleaved OPN levels in SF according to the genotypes of the OPN gene polymorphisms. Our results showed that the thrombin-cleaved OPN levels in SF were significantly lower in -443TT carriers compared with the -443TC and -443CC carriers (4,728 ± 354 vs. 5,398 ± 354 and 5334 ± 323, pg/ml, both *P *< 0.05). Similarly, the thrombin-cleaved OPN levels in SF from -66GG were lower than from the -66GT and -66TT carriers (4,989 ± 329 vs. 5,413 ± 357 and 5,426 ± 274, pg/ml, both *P *< 0.05). The genetic polymorphisms of -156G/GG did not influence the thrombin-cleaved OPN levels in SF (Figure 2). Western blot results showed that the thrombin-cleaved OPN expressions in the SF from -443TT patients were markedly lower than that from the -443TC and -443CC patients. The thrombin-cleaved OPN expressions in the SF from -66 GG patients were lower than those from -66GT and -66TT carriers (Figure 3). The protein expression of full-length OPN levels in SF were similar among -443TT carriers compared with the -443TC and -443CC, as well as the -66GG, -66GT and -66TT genotype carriers (figure not shown).

## Discussion

In this study, we investigated the role of *OPN *gene polymorphisms in determining the susceptibility to, and radiographic severity of OA in a Chinese cohort. Of three gene polymorphisms in this study, we found the polymorphisms of two loci, namely, the -443C/T and the -66T/G, were significantly associated with the risk and radiographic severity of OA. The -443TT and -66GG polymorphisms showed a protective effect against occurrence of OA and were associated with lower radiographic severity. In addition, the polymorphisms of -443C/T and -66T/G significantly affected the thrombin-cleaved OPN levels in SF from OA patients. Patients with the -443TT and -66GG genotypes had markedly lower thrombin-cleaved OPN levels in SF than those with -443CT, -443CC and -66 GT and -66TT. Moreover, the thrombin-cleaved OPN levels in SF were positively correlated with the radiographic severity of OA. To the best of our knowledge, this is the first study of the significance of *OPN *gene polymorphisms in the susceptibility to, and severity of OA. Our findings suggest that certain *OPN *gene polymorphisms may be used as molecular markers for susceptibility to, and severity of OA.

OPN is an extracellular matrix protein with pleiotropic properties, and has been recently recognized as a potential inflammatory cytokine [27]. Expression of OPN has been observed in the joints of patients with rheumatoid arthritis (RA) as well as OA [28]. The function of OPN is modulated by protease digestion, and a thrombin-cleaved form of OPN is involved in the pathogenesis of various inflammatory disorders, including OA and RA [29,25,30].

More than fifty single nucleotide polymorphisms have been identified in the human *OPN *encoding gene in different populations [31]. We selected three SNPS based on previous association with autoimmune and/or inflammatory diseases and ability to determine haplotypes predicting serum OPN concentrations [32,21,34]. The SNP at -66 was predicted to bind the specificity protein 1 (SP1) transcription factor, as it is known that the sequence between -68 and -59 is an SP1 binding site in the human OPN promoter [35]. A previous study showed that SP1 and SP3 factors recognize this site and that the allele with the -66T nucleotide inside the SP1 recognition sequence has a higher binding affinity [31]. Among patients with cancer, those with G/G at -156 have been found to have higher concentrations of OPN than those with G/GG or GG/GG. Patients with G/G at -156 have more frequently been diagnosed with advanced stage than with early stage non-small cell lung cancer (NSCLC) [36]. Another study revealed that the functional -443C/T polymorphism influences *OPN *gene expression in melanoma cells via binding of c-Myb transcription factor [37]. To date, these genetic variations of the *OPN *gene have been described to be associated with inflammatory diseases, including lupus erythematosus, multiple sclerosis, urolithiasis, primary biliary cirrhosis, and autoimmune lymphoproliferative syndrome.

Our results in this study showed that the thrombin-cleaved OPN levels in SF were significantly lower in -443TT compared with -443TC and -443CC carriers. A previous study examined the effect of the -443T/C polymorphism on transcription of the *OPN *gene. The authors measured promoter activity with a dual luciferase reporter assay system and compared the activities of the -443C and -443T alleles with the MKN28 and SGC-7901 cell lines. They found significantly higher luciferase activities were generated with the pGL3-C construct compared to the pGL3-T construct [38]. A recent study of metastases in melanoma found that those homozygous for the -443C allele expressed significantly higher levels of OPN mRNA compared to those that were either heterozygous (CT) or homozygous for the -443T allele. Transcription factor c-Myb binds to the region of the *OPN *promoter in an allele-specific manner and induces enhanced activity of the -443C compared to the -443T *OPN *promoter [37]. These studies provide information on the mechanism under which the -443T/C polymorphism regulates the *OPN *gene transcript activity, thus influencing OPN expression.

A study reported that the -66 polymorphism modifies the binding affinity for the SP1/SP3 transcription factors. The authors found that SP1 and SP3 factors recognize this site and that the allele with the T nucleotide inside the SP1 recognition sequence has a higher binding affinity. Overexpression of SP1 induced higher promoter activation on a plasmid carrying the T allele compared with the equivalent plasmid carrying the G allele. Consistent with this finding, we found the thrombin-cleaved OPN levels in SF from -66GG was lower than -66GT and -66TT.

Two previous studies investigated the association between the *OPN *gene polymorphisms and RA. Unlike the reported effect of the *OPN *SNP conferring predisposition to common diseases such as multiple sclerosis or systemic lupus erythematosus, four SNPs of the *OPN *gene (327T/C, 795C/T, 1128A/G, and 1284A/C) did not contribute to RA susceptibility in a Spanish population [39]. Another study in a Chinese population showed that *OPN *gene polymorphisms do not correlate with susceptibility to RA [40]. In the present study, we found that the -443C/T and -66T/G were associated not only with risk of OA, but also its radiographic severity, suggesting the role of *OPN *genetic polymorphisms in OA and RA may be quite different.

Elevated levels of OPN have been found in SF from RA patients and increased levels of OPN have been correlated with increased levels of multiple inflammatory cytokines [28]. A recent study showed that thrombin-cleaved OPN levels from SF samples correlate with radiographic severity determined by KL grading of knee OA in a Japanese study [29]. In that study, immunohistochemistry of the synovium showed stronger reactivity in samples from subjects with advanced OA. Consistent with this finding, we observed in the present study that thrombin-cleaved OPN levels from SF were significantly higher in those with KL grade > 3 than in those with KL grade = < 3; by stratifying the thrombin-cleaved OPN levels according to the OPN genotypes, we found that the -443 and -66 polymorphisms had a substantial influence on thrombin-cleaved OPN levels. The -443TT and -66GG, which showed a protective role against susceptibility to OA, had markedly lower thrombin-cleaved OPN levels. Our data confirmed the positive correlation between thrombin-cleaved OPN levels in SF and OA severity. We suggest that thrombin-cleaved OPN may serve as a biochemical marker for determining disease severity and could be predictive of prognosis with respect to the progression of knee OA. Since previous studies showed that the alleles at polymorphic sites in the *OPN *gene influence the OPN level by influencing the level of promoter activity and characterizing their ability to bind transcription factors, we here postulated that the influence of *OPN *polymorphisms on risk and severity of OA is very likely due to its effect on the synthesis of the active OPN protein.

Some limitations of this study need to be addressed. First, there was a large proportion of obese patients in this study. As obesity is an important risk factor for OA, not only as a mechanical factor but also due to the related increase in cytokines, which contribute to OA development. Thus, there might have been enrollment bias in the recruitment of patients with primary OA into this study. Second, we enrolled a Chinese cohort of patients with knee OA only, so, whether the positive roles of OPN gene polymorphisms we found in this study exist in other types of OA, or in other ethnic populations, remains unknown. Third, we did not perform functional analyses to further disclose the mechanism under which the *OPN *gene polymorphisms affect the susceptibility to and severity of OA.

## Conclusions

In this study we found the polymorphisms of two loci of OPN gene, namely, the -443C/T and the -66T/G, were significantly associated with risk and radiographic severity of OA, suggesting that these *OPN *gene polymorphisms may be used as molecular markers for the susceptibility to, and severity of OA.

## Abbreviations

BMI: body mass index; CV: coefficient of variation; ELISA: enzyme-linked immunosorbent assay; GAPDH: glyceraldehyde-3-phosphate dehydrogenase; KL: Kellgren-Lawrence; NSCLC: non-small cell lung cancer; OA: osteoarthritis; OPN: osteopontin; OR: odds ratio; PBS: phosphate-buffered saline; RA: rheumatoid arthritis; SF: synovial fluid; SP: specificity protein.

## Competing interests

The authors declare that they have no competing interests.

## Authors' contributions

YJ: study design, manuscript writing; MY, CZ and QL: OPN genotyping and clinical data collection. All authors have read and approved the manuscript for publication.
